# OP65 Focusing On What Matters Most: A Public Dialogue On How NICE Should Prioritize Topics In Health Technology Assessment

**DOI:** 10.1017/S0266462324001259

**Published:** 2025-01-07

**Authors:** Alice Murray, Koonal Shah

## Abstract

**Introduction:**

To meet the needs of an evolving health and care system, the National Institute of Health and Care Excellence (NICE) is changing its approach to topic prioritization so it can focus on what matters most. To support this, NICE ran a public dialogue to gather informed opinion on how it should select topics for guidance, including for some technology evaluation programs.

**Methods:**

Fifty-five general public participants from across England took part in two face-to-face and three online deliberative workshops (each lasting two or three hours, held over four weeks in 2023). Participants were asked to consider the following criteria in the context of prioritization: health and care need, evidence availability, system impact, budget impact, health inequalities, and environmental sustainability. The workshops were designed to understand whether any aspects were more important than others and explore the reasons why. They used deliberative engagement methods and included trade-off exercises, role-play, group discussion, ranking tasks, and interactions with specialists.

**Results:**

Emerging findings show that the participants think NICE should consider several aspects when prioritizing topics for guidance. Health and care need was of primary importance for people, followed by evidence availability, budget impact, and system impact. Health inequalities and environmental sustainability were generally considered to be less important, though participants still felt these were areas that should inform NICE’s prioritization decisions. Participants identified relevant interactions between the criteria, suggesting that each criterion cannot be considered in isolation. Full results will be available to present at HTAi 2024.

**Conclusions:**

Deliberative public engagement is a meaningful way to involve the public in complex policy decisions with a social value element. Broad public agreement was found with the criteria NICE has proposed to consider when prioritizing topics for guidance, and some criteria are more important to people than others. The findings will feed into NICE’s new approach to topic prioritization.

